# Complete Genome Sequence of a Pseudomonas aeruginosa Isolate from a Kidney Stone

**DOI:** 10.1128/MRA.01073-19

**Published:** 2019-09-19

**Authors:** Genevieve Johnson, Nicole Stark, Alan J. Wolfe, Catherine Putonti

**Affiliations:** a Bioinformatics Program, Loyola University Chicago, Chicago, Illinois, USA; b Department of Biology, Loyola University Chicago, Chicago, Illinois, USA; c Department of Microbiology and Immunology, Stritch School of Medicine, Loyola University Chicago, Maywood, Illinois, USA; d Department of Computer Science, Loyola University Chicago, Chicago, Illinois, USA; University of Arizona

## Abstract

Recently, we isolated a temperate bacteriophage, *Pseudomonas* phage Dobby, from a calcium oxalate kidney stone. Here, we present the complete genome of the bacterial host harboring this phage, Pseudomonas aeruginosa UMB2738. From the analysis of the genome sequence, five additional prophage sequences were identified.

## ANNOUNCEMENT

While bacteria are the cause of magnesium-ammonium-phosphate (struvite) kidney stones (1), they may also contribute to calcium kidney stones (2–5). Studies have successfully cultured bacteria from kidney stones (2–8) and have most frequently isolated Escherichia coli and Pseudomonas species (9). In a recent study by Barr-Beare et al. (5), E. coli and Pseudomonas aeruginosa were cultured from two calcium oxalate (CaOx) stones. From one of these stones from a male patient, two P. aeruginosa colonies, identified via 16S rRNA gene sequencing, were isolated (5). In one of these isolates, UMB2738, the φCTX-like temperate *Pseudomonas* phage Dobby was isolated and characterized (10). Similarly, a temperate phage was induced from the other P. aeruginosa isolate, UMB2744. This prompted us to sequence both of these bacterial isolates to ascertain first if they belonged to the same strain and, subsequently, to characterize P. aeruginosa from CaOx stones.

P. aeruginosa strains UMB2738 and UMB2744 were isolated and cultured using the expanded quantitative urinary culture (EQUC) protocol (11) as described by Barr-Beare et al. (5) and stored at −80°C. From this freezer stock, each isolate was streaked onto a 1.7% LB agar plate and grown overnight at 37°C. A single colony from each plate was selected and grown in LB liquid medium overnight at 37°C with shaking with 5-mm sterile glass beads to minimize biofilm formation. DNA was extracted using the Qiagen DNeasy UltraClean microbial kit and quantified using a Qubit fluorometer. DNA was sent to the Microbial Genomic Sequencing Center (MiGS) at the University of Pittsburgh for sequencing. Briefly, DNA was enzymatically fragmented using an Illumina tagmentation enzyme, and indices were attached using PCR. The barcoded samples were multiplexed on an Illumina NextSeq 500 flow cell, producing 1,053,541 and 943,073 pairs of 151-bp reads for UMB2738 and UMB2744, respectively. Default parameters were used for all software tools unless otherwise noted. Raw reads were trimmed using Sickle v1.33 (https://github.com/najoshi/sickle) and then assembled using SPAdes v3.13.0 (parameters, only-assembler; k = 55,77,99,127) (12). The assembled contigs were concatenated for each sample and compared via the progressiveMauve algorithm v1.1.1 (13) in Geneious Prime (Biomatters Ltd., Auckland, New Zealand). This comparison revealed that UMB2738 and UMB2744 had identical genomes (pairwise identity = 100%). Thus, the genome was reassembled with all 13,523,760 trimmed reads from both barcodes using SPAdes and the same parameters. Here, we refer to this strain as UMB2738.

The genome assembly of P. aeruginosa UMB2738 includes 87 contigs and consists of 6,720,267 bp with a GC content of 66.15%. The genome coverage is 79.35×, calculated by BBMap v38.47 (https://sourceforge.net/projects/bbmap/), and the assembly has an *N*_50_ score of 135,797. Genome annotation was performed using the NCBI Prokaryotic Genome Annotation Pipeline (PGAP) v4.8 (14). No CRISPR/Cas genes or arrays were found within the genome by CRISPRCasFinder (15). The genome encodes 6,298 protein coding genes, three rRNA operons, and 57 tRNAs. As this strain was the source of the temperate *Pseudomonas* phage Dobby (10), the genome assembly was queried via blastn (16) for the Dobby sequence (GenBank accession number MK034952) and located in the first, largest contig of the assembly. The P. aeruginosa UMB2738 genome was also screened for phage sequences using VirSorter v1.0.5 (17). Six prophage sequences were predicted, including the region encoding Dobby. VirSorter also identified *Pseudomonas* phage Pf1 and a relative of *Pseudomonas* phage vB_Pae_CF79a (GenBank accession number MK510971; query coverage, 45%; identity, 97.18%). The other two phage sequences predicted do not exhibit any sequence homology to characterized phage sequences.

Sequencing of this bacterial isolate will inform our future work with *Pseudomonas* phage Dobby, which is just one of the phages harbored by this bacterium. Furthermore, the availability of this genome, representative of bacteria within kidney stones, can add insight into future studies of the urinary tract microbiota.

### Data availability.

This whole-genome shotgun (WGS) project has been deposited in GenBank under the accession number VSIZ00000000. Raw sequence reads are deposited under accession numbers SRR9992785 and SRR9992786. The WGS and SRA records are associated with BioProject number PRJNA316969.
